# Avoidance of Trinucleotide Corresponding to Consensus Protospacer Adjacent Motif Controls the Efficiency of Prespacer Selection during Primed Adaptation

**DOI:** 10.1128/mBio.02169-18

**Published:** 2018-12-04

**Authors:** Olga Musharova, Danylo Vyhovskyi, Sofia Medvedeva, Jelena Guzina, Yulia Zhitnyuk, Marko Djordjevic, Konstantin Severinov, Ekaterina Savitskaya

**Affiliations:** a Center for Life Sciences, Skolkovo Institute of Science and Technology, Moscow, Russia; b Institute of Molecular Genetics, Russian Academy of Sciences, Moscow, Russia; c Institute of Physiology and Biochemistry, Faculty of Biology, University of Belgrade, Belgrade, Serbia; d Waksman Institute, Rutgers, The State University of New Jersey, Piscataway, New Jersey, USA; University of Melbourne; Rockefeller University; University of Montana

**Keywords:** CRISPR spacers, CRISPR-Cas, naïve adaptation, primed adaptation

## Abstract

CRISPR DNA arrays of unique spacers separated by identical repeats ensure prokaryotic immunity through specific targeting of foreign nucleic acids complementary to spacers. New spacers are acquired into a CRISPR array in a process of CRISPR adaptation. Selection of foreign DNA fragments to be integrated into CRISPR arrays relies on PAM (protospacer adjacent motif) recognition, as only those spacers will be functional against invaders. However, acquisition of different PAM-associated spacers proceeds with markedly different efficiency from the same DNA. Here, we used a combination of bioinformatics and experimental approaches to understand factors affecting the efficiency of acquisition of spacers by the Escherichia coli type I-E CRISPR-Cas system, for which two modes of CRISPR adaptation have been described: naive and primed. We found that during primed adaptation, efficiency of spacer acquisition is strongly negatively affected by the presence of an AAG trinucleotide—a consensus PAM—within the sequence being selected. No such trend is observed during naive adaptation. The results are consistent with a unidirectional spacer selection process during primed adaptation and provide a specific signature for identification of spacers acquired through primed adaptation in natural populations.

## Abstract (precis)

Adaptive immunity of prokaryotes depends on acquisition of foreign DNA fragments into CRISPR arrays as spacers followed by destruction of foreign DNA by CRISPR interference machinery. Different fragments are acquired into CRISPR arrays with widely different efficiencies, but the factors responsible are not known. We analyzed the frequency of spacers acquired during primed adaptation in an E. coli CRISPR array and found that AAG motif was depleted from highly acquired spacers. AAG is also a consensus protospacer adjacent motif (PAM) that must be present upstream from the target of the CRISPR spacer for its efficient destruction by the interference machinery. These results are important because they provide new information on the mechanism of primed spacer acquisition. They add to other previous evidence in the field that pointed out to a “directionality” in the capture of new spacers. Our data strongly suggest that the recognition of an AAG PAM by the interference machinery components prior to spacer capture occludes downstream AAG sequences, thus preventing their recognition by the adaptation machinery.

## INTRODUCTION

Prokaryotic CRISPR-Cas systems consisting of CRISPR arrays containing identical repeats separated by unique spacers and associated *cas* genes protect cells from invading nucleic acids (1–3). CRISPR-Cas systems function by first acquiring fragments of invading nucleic acids, prespacers, and integrating them into CRISPR arrays as spacers, thus forming hereditable immunological memory (4). DNA of genetic invaders containing “memorized” fragments is recognized by Cas protein complexes and spacer-containing CRISPR RNAs (crRNAs) and targeted for destruction in a process called CRISPR interference (5). The recognition is achieved through complementary interaction between crRNA spacer and the target sequence, named the protospacer, and is also dependent on a specific short protospacer adjacent motif (PAM) (6–10).

CRISPR-Cas systems developed diverse mechanisms to avoid autoimmunity that should arise from targeting spacers in CRISPR array. Most of these mechanisms are based on a requirement for PAM, which is not complementary to crRNA but is specifically recognized by Cas proteins from the interfering complex (11, 12). The PAM is absent from the CRISPR repeat sequence adjoining the spacer. The separation of CRISPR defense into spacer acquisition and target interference stages and the requirement for PAM means that new spacers need to arise from sequences (prespacers) associated with PAM. Otherwise, they will not be able to perform their protective function.

For a well-studied type I-E CRISPR-Cas system from Escherichia coli, two modes of spacer adaptation have been described (13–15). The naive adaptation requires the Cas1 and Cas2 proteins and a CRISPR array (15). About 40% of spacers acquired during the naive adaptation arise from prespacers associated with the consensus AAG PAM; the majority of other acquired spacers are not expected to be functional in interference (15). In addition to Cas1 and Cas2, primed adaptation requires all the components of the interference stage: in E. coli they are the complex Cascade, the Cas3 nuclease-helicase, and a crRNA, which recognizes foreign DNA (13). Primed adaptation is much more efficient than naive adaptation, and almost 100% of prespacers chosen contain a consensus AAG PAM (16). The requirement for specific crRNA indicates that primed adaptation is triggered by the recognition of the target by the Cascade-crRNA effector complex. The site of recognition is referred to as a “priming protospacer.” Upon target recognition by the effector complex, localized melting of the protospacer occurs. Melting initiates close to the PAM, in the so called “seed” region of the protospacer, and then extends further downstream (17). One protospacer strand, referred to as the “target strand,” forms a heteroduplex with crRNA spacer sequence. The other, nontarget, strand is displaced, forming an R-loop. A specific feature of primed adaptation is a very strong strand bias in the orientation of selected prespacers (13, 14, 16). Upstream of the priming site, more than 90% of prespacers are oriented the same way as the priming protospacer: i.e., they map on the nontarget strand. The orientation of downstream prespacers is an opposite one: i.e., they map to the target strand. The efficiency of prespacer acquisition decreases with increasing distance from the priming site (18). No such biases are apparent during naive adaptation, and acquired spacers map to both strands of foreign DNA. It was shown that naive adaptation is affected by RecBCD activity, and acquired spacers tend to originate from regions with double-stranded breaks or replication fork stalling (19, 20).

While the presence of an AAG PAM at a prespacer side is strictly required for its selection by the adaptation machinery during primed adaptation and makes a strong contribution during naive adaptation, it alone does not determine the efficiency of prespacer usage (21, 22). Thus, in an E. coli culture undergoing primed adaptation of spacers from a plasmid, it is commonly observed that certain prespacers with an AAG PAM are acquired by many cells, while others are acquired rarely or not at all (22). The former are referred to as “hot” prespacers, while the latter are “cold.” The pattern of hot and cold prespacers and their relative efficiencies are highly reproducible. The reasons behind the observed differential use of prespacers during adaptation are not known. In this work, we performed bioinformatics and experimental analysis that led us to conclude that a presence of an AAG trinucleotide within the prespacer has a strong negative effect on the frequency of its use during primed adaptation.

## RESULTS

### Spacers efficiently acquired during primed adaptation have distinct nucleotide composition.

**FIG 1:**
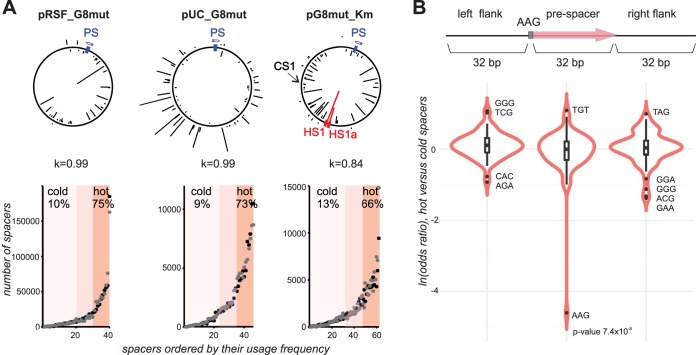
Prespacers actively used during primed adaptation are depleted in the AAG trinucleotide. (A) At the top, a graphical representation of spacers acquired in the course of primed adaptation from plasmids pRSF_G8mut, pUC_G8mut, and pG8mut_Km is presented. The position of the priming protospacer G8 (PS) in each plasmid is indicated by a blue rectangle. Arrows indicate the orientation of the priming protospacer (same in pRSF_G8mut and pG8mut_Km and opposite in pUC_G8mut). Spacers acquired from each plasmid are shown by black lines, with line heights indicating relative frequency of reads corresponding to different spacers. Lines projecting inside and outside the plasmid circles represent spacers mapping on opposite strands of plasmid DNA. Spacers originating from hot spot 1 (HS1) and HS1a prespacers (see the text for details) are highlighted in red. “CS1” shows the position of the cold prespacer (see the text for details). Below, Pearson correlation coefficients for mapping of spacers acquired from each plasmid in two independent experiments are given. At the bottom, spacers acquired from each plasmid were ranked according to their occurrence in Illumina reads. Each dot represents one spacer (corresponding to lines protruding from plasmid maps at the top). Dots colored black and gray represent results from two independent experiments. Spacers in the lower half of the distribution were considered cold. The top 25% of most common spacers were considered hot. The mean total percentage of cold and hot spacers from two experiments for each plasmid is given. (B) Violin plots showing odds ratio of trinucleotides in hot versus cold prespacers and their flanking sequences. The *P* value for AAG depletion in hot prespacers is shown.

To reveal possible causes of unequal acquisition efficiency of prespacers during primed adaptation, previously reported data sets of spacers acquired by E. coli KD263 cells transformed with plasmids pRSF_G8mut and pUC_G8mut (23, 24) were analyzed (see Table S1 in the supplemental material). In addition, new data sets of spacers acquired by KD263 cells in the presence of pG8mut_Km plasmid (Materials and Methods) were used. In each case, adaptation was initiated from a plasmid-borne G8mut priming protospacer partially matching the spacer segment of KD263 crRNA. The backbones of pRSF_G8mut, pUC_G8mut, and pG8mut-Km are sufficiently different so that most spacers of each data set do not overlap. For each sample, data sets corresponding to two biological replicates were analyzed. As expected for primed adaptation, most spacers in each culture were acquired from plasmid (99.7%) rather than the bacterial genome, and 86.35% of plasmid-derived spacers mapped to the DNA strand that was not targeted by G8 crRNA (Fig. 1A; Table S1). A total of 98.4% of plasmid spacers originated from prespacers preceded by an AAG PAM. The distribution of frequencies of spacers was highly reproducible for each plasmid, with a Pearson correlation of 0.84 or higher. While it has been observed that regions proximal to a priming protospacer preferentially donate new spacers during primed adaptation (18, 25–27), there was no gradient in prespacer usage with any of the plasmids (Fig. 1A), likely due to their small size.

10.1128/mBio.02169-18.3 TABLE S1 Statistics for spacers acquired during primed adaptation. Download Table S1, DOCX file, 0.1 MB. Copyright © 2018 Musharova et al. 2018 Musharova et al. This content is distributed under the terms of the Creative Commons Attribution 4.0 International license.

For each plasmid, sequences of unique spacers derived from the nontarget strand and associated with AAG PAM were sorted according to spacer frequency in the data set. The resulting frequency distributions for each plasmid are shown in Fig. 1A. As can be seen, the distributions are highly unequal, with some spacers being used much more frequently than others. We consider the 25% of most frequently used spacers as “hot.” Conversely, 50% of spacers at the opposite end of the distribution are considered “cold.” Together, sequences from the hot spacer group account for ∼70% of all plasmid-borne spacers, while cold spacer group sequences account for ∼10% of spacers. For subsequent analysis, unique hot and cold group spacers from each data set were combined and treated together.

No difference in nucleotide composition of “cold” and “hot” spacers was revealed. Dinucleotide frequency analysis was likewise uninformative (data not shown). Strikingly, analysis of trinucleotide frequencies showed that the AAG triplet was strongly underrepresented in the hot group (Fig. 1B) (*P* = 7.4 × 10^−8^).

We also considered whether sequences flanking plasmid prespacers have an effect on prespacer acquisition frequency during primed adaptation. Spacer-sized 33-bp regions upstream of AAG PAMs or downstream of “hot” and “cold” prespacers were also analyzed, but no strong bias was detected in either base composition or di/trinucleotide frequencies (see Fig. 1B for trinucleotide frequency).

### The presence of the AAG trinucleotide within a prespacer controls the efficiency of its use as a donor of spacers during primed adaptation.

**FIG 2:**
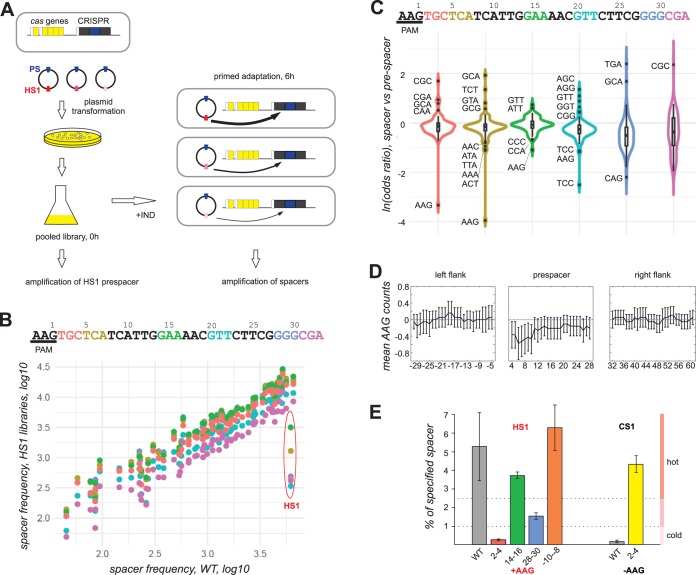
Experimental demonstration of position-specific AAG avoidance in hot prespacers during primed adaptation. (A) A workflow of the library-based approach to determine the effect of prespacer sequence on acquisition efficiency is presented. Engineered E. coli KD263 cells with inducible expression of *cas* genes and a CRISPR array with a single G8 spacer are transformed with a library of plasmids containing the G8 priming protospacer (blue) and randomized trinucleotides in the HS1 prespacer (shown by different hues of red); white rectangles represent promoter regions of *cas* genes and the CRISPR array. Transformants grown on selective medium are pooled and placed in a medium without antibiotic required for plasmid maintenance. The cultures are induced and grown for 6 h to allow primed adaptation to occur. In the pooled culture before induction, the HS1-containing region is amplified and subjected to Illumina sequencing. In the induced culture, the CRISPR array is amplified, and amplicon corresponding to expanded array is subjected to Illumina sequencing. (B) At the top, the sequence of the HS1 prespacer and its PAM is shown. Trinucleotides subjected to randomization in six different libraries are indicated by colors. Below, the frequency of spacers acquired by cells carrying each library is compared to the frequency of spacer acquisition in the initial plasmid (WT). Each dot represents a spacer, and the color of the dot corresponds to the color of the randomized trinucleotide. Dots corresponding to HS spacer and its variants are indicated. (C) Violin plots showing odds ratio of trinucleotides in HS1-derived spacers compared to prespacers in each library. (D) The left, middle, and right plots correspond, respectively, to 33 bp of upstream prespacer flank, the prespacer sequence, and the downstream prespacer flank. Coordinates on the *x* axis correspond to the center of the 6-bp sliding window, where +1 corresponds to G in AAG PAM. The difference between mean AAG counts in hot and cold prespacer categories is shown in the *y* axis. The error bars correspond to 95% confidence intervals. (E) Acquisition of HS1 and CS1 spacer variants from individual plasmids carrying trinucleotide substitutions. The bars show the percentage of HS1 and its variants and CS1 and its variant to overall plasmid-derived spacers acquired by cells carrying wild-type pG8mut_Km (WT) or derivatives carrying AAG trinucleotides at specified positions of HS1 or carrying an AAC trinucleotide instead of AAG at positions 2 to 4 of the CS1 prespacer. Mean values obtained from two independent experiments and standard deviations are given.

To experimentally measure the contribution of nucleotide sequence to spacer acquisition efficiency, we studied the effects of sequence alterations in HS1 (hot spot 1), one of the most commonly used hot prespacers from the pG8mut-Km plasmid (Fig. 1A). The acquisition of this prespacer was analyzed previously, and it was shown that its usage depends on the AAG PAM (22). Six pG8mut-Km plasmid libraries containing randomized trinucleotides at HS1 positions 2 to 4, 5 to 7, 14 to 16, 20 to 22, 28 to 30, and 31 to 33 were prepared. Each library was transformed in uninduced KD263 cells, and pooled transformants were subjected to PCR with a pair of primers annealing upstream and downstream of plasmid region spanning the HS1 prespacer (Fig. 2A). Analysis of Illumina reads from obtained amplicons revealed that for each library, all 64 expected variants were present.

For each library, several thousand transformants were pooled and grown in the presence of inducers of *cas* gene expression in the absence of antibiotic. These conditions stimulate primed adaptation from the plasmid without selecting against cells that acquired interference-proficient spacers targeting the plasmid. Amplified DNA fragments corresponding to the expanded CRISPR array in cultures harboring each plasmid library were subjected to Illumina sequencing, and acquired spacers were analyzed. The overall pattern of plasmid-derived new spacers was the same in each library and matched the one observed for unmodified pG8mut-Km (Fig. 2B). The only exception were spacers corresponding to HS1, whose cumulative efficiency of adaptation decreased in the libraries compared to unmodified pG8mut-Km. Sequences of acquired spacers matching HS1 and its variants were extracted, and odds ratios between frequency of spacer variants and prespacer variants in corresponding libraries were determined. As can be seen from results presented in Fig. 2C, HS1 spacer variants with the AAG trinucleotide in the seed region (positions 2 to 4 and 5 to 7) were strongly underrepresented. The effect was much weaker at positions 14 to 16, 20 to 22, 28 to 30, and 31 to 33. We conclude that the library approach supports the bioinformatics analysis that shows that the presence of internal AAG inhibits prespacer usage during primed adaptation. The results also show that the effect is position specific and is most evident when the AAG trinucleotide is located in the seed of the future spacer.

Given the observed position specificity of library data, we reanalyzed hot spacers from the combined plasmid set (Fig. 1B) using a 6-base sliding window and concentrating on comparison of the 10% “hottest” and “coldest” spacers. The results, presented in Fig. 2D, confirmed the avoidance of AAG in the seed region of these spacers. The remaining positions exhibited a bias of marginal statistical significance, while no bias was observed in spacer-sized flanking sequences upstream or downstream of hot prespacers. The positional bias in AAG occurrence was also revealed using an independent approach, by analyzing the entire spacer set and correlating AAG counts in different prespacer regions and the corresponding spacer frequencies (see Fig. S1 in the supplemental material).

10.1128/mBio.02169-18.1 FIG S1 The correlation between AAG counts and observed spacer frequencies. The *x* axis indicates 3 nonoverlapping 11-bp prespacer regions (upstream, middle, and downstream). For each of these regions, Pearson’s coefficient of correlation (*R*) between AAG counts and observed spacer frequencies is shown. Error bars correspond to 95% confidence intervals, and the corresponding *P* values are added below each bar. The figure shows that AAG presence has the strongest (and statistically highly significant) negative effect on the acquisition frequency in the upstream segment, although a statistically significant negative effect is also observed for the other two regions. Download FIG S1, TIF file, 14.5 MB. Copyright © 2018 Musharova et al. 2018 Musharova et al. This content is distributed under the terms of the Creative Commons Attribution 4.0 International license.

To directly demonstrate that the presence of AAG trinucleotide affects prespacer acquisition, individual plasmids containing AAG at HS1 positions 2 to 4, 14 to 16, and 28 to 30 were constructed and used in a primed adaptation experiment. Analysis of spacers acquired by cells carrying these plasmids revealed that compared to pG8mut-Km, the presence of AAG at positions 2 to 4 decreased the number of HS1-derived spacers more than 10 times (Fig. 2E). Introduction of AAG at positions 14 to 16 and 28 to 30 had a milder, 2- to 3-fold effect. When an AAG trinucleotide was introduced 5 nucleotides upstream of HS1 PAM, no effect on HS1 spacer acquisition efficiency was detected.

We also determined whether removal of an AAG trinucleotide increases the usage of a cold prespacer. The pG8mut-Km prespacer CS1 (cold spot 1) contains an AAG at positions 2 to 4. When substituted for AAC, the use of this prespacer increased ∼16-fold, placing it in a hot spacer group.

### The presence of AAG trinucleotide has no effect on prespacer usage during naive adaptation.

**FIG 3:**
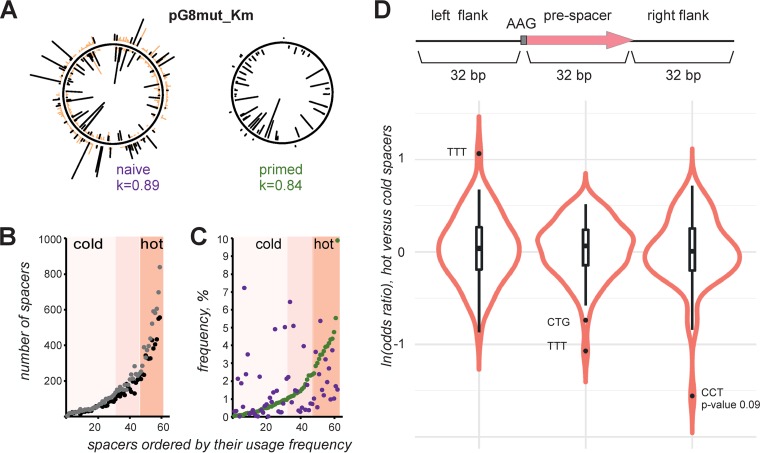
Comparison of prespacers acquired during naive and primed adaptation. (A) At the top, a graphical representation of spacers acquired in the course of naive (left) and primed (right) adaptation from the pG8mut_Km plasmid is presented. See the legend to Fig. 1A for details. For naive adaptation, spacers mapping to prespacers with the AAG PAM are shown by black lines. Spacers mapping to prespacers with non-AAG PAMs are marked in orange. (B) Spacers acquired during naive adaptation (A) that mapped to prespacers with the AAG PAM and the “inner” strand of plasmid DNA were ranked according to their occurrence in Illumina reads. Each dot represents one spacer (which corresponds to lines protruding from the plasmid map in panel A, left). Dots colored black and gray represent results from two independent experiments. Spacers in the lower half of the distribution were considered cold. The top 25% of most common spacers were considered hot. (C) Spacers acquired from pG8mut_Km in the course of primed adaptation were ranked as in Fig. 1A: each spacer is represented by a green dot. The frequency of corresponding spacers acquired in the course of naive adaptation is represented by dark violet dots. (D) Violin plots showing odds ratio of trinucleotides in hot versus cold prespacers and their flanking sequences from the naive adaptation experiment.

We were interested in comparing prespacer choice preferences during primed and naive adaptation. The “naive” spacer set was obtained by transforming the pG8mut-Km plasmid in E. coli BL21(DE3) cells carrying a compatible plasmid coexpressing the Cas1 and Cas2 proteins. BL21(DE3) lacks its own *cas* operon, and in the presence of pCas1 + 2 is only capable of naive adaptation (15). Mapping of spacers acquired in the BL21(DE3) CRISPR array from pG8mut-Km is shown in Fig. 3A (left-hand side). As expected, there was no strand bias and many spacers originated from prespacers without AAG PAM (see Table S2 in the supplemental material). The pattern of spacers acquired during naive adaptation (Fig. 3A, left-hand side) is highly reproducible (Pearson coefficient of 0.89) and distinct from the pattern of spacers acquired from pG8mut-Km during primed adaptation (shown on the right-hand side of Fig. 3A). To compare prespacer preferences during two modes of adaptation, we concentrated on prespacers with an AAG PAM mapping to the “inner” strand of pG8mut-Km, as shown in Fig. 3A. The efficiency of usage of such prespacers during naive adaption (ranked according to increasing occurrence of spacers as in Fig. 1B) is shown in Fig. 3B for two independent experiments. On Fig. 3C, frequencies of spacers from the naive set are plotted alongside the ranked set of spacers acquired during primed adaptation. Visual inspection of data and statistical analysis show that there is no correlation between the two sets (Pearson correlation of 0.19; *P* value of 0.14). In other words, a spacer that scores as cold (or hot) during primed adaptation can be either cold or hot or have intermediate frequency during naive adaptation.

10.1128/mBio.02169-18.4 TABLE S2 Statistics for spacers acquired during naive adaptation. Download Table S2, DOCX file, 0.1 MB. Copyright © 2018 Musharova et al. 2018 Musharova et al. This content is distributed under the terms of the Creative Commons Attribution 4.0 International license.

Since the sets of hot and cold spacers in naive and primed adaptation are distinct, we wondered if any specific sequence signatures can be revealed in spacers that were acquired during naive adaptation with different efficiencies. For this analysis, unique spacers acquired from pG8mut-Km and the pCas1 + 2 plasmid coexpressing *cas1* and *cas2* were combined into a single set and analyzed jointly. However, no specific signal for single nucleotides, dinucleotides, and trinucleotides was observed. Consistent with results shown in Fig. 3C, the frequency of spacers acquired from prespacers associated with AAG PAM during naive adaptation was not affected by the presence of the internal AAG trinucleotide (Fig. 3D). Similar to observations with primed adaptation, upstream and downstream flanking sequences contained no specific features.

## DISCUSSION

Spacer diversity in CRISPR arrays from native bacterial strains is very high (28). Spacer selection is nonrandom, and strong and reproducible biases in acquired spacer repertoires were described for both naive and primed adaptation in laboratory experiments (16, 21, 29–32). While such biases can be produced by selection for spacers most efficient during CRISPR interference, preferences of the adaptation machinery must also play a role. Understanding the determinants of efficient spacer acquisition in the absence of selection may be useful for designing experiments in which adapted spacers are used to record cellular events in the absence of subsequent interference (29, 30). In this work, we compared the efficiency of prespacer selection by the E. coli type I-E CRISPR-Cas system during primed and naive adaptation in the absence of selection. Earlier analysis of efficiently acquired spacers during naive adaptation in this system revealed that actively used prespacers may contain motifs in their 3′ ends. However, these motifs appear to be mutually exclusive (AA at positions 32 and 33 according to Yosef et al. [21], compared to G at position 32 in the study by Shipman et al. [30]). In the case of primed adaptation by I-C and I-B CRISPR-Cas systems, it has been shown that nucleotide substitutions in the prespacer affect the efficiency of its use (31, 32). Overall, these earlier works show that prespacer sequence clearly contributes to its selection efficiency during adaptation. Our analysis failed to reveal determinants of prespacer naive adaptation efficiency. However, we observed very strong avoidance of AAG trinucleotide in spacers efficiently acquired during primed adaptation. The AAG trinucleotide is also the dominant (99.8%) PAM of prespacers that are acquired during primed adaptation. The complementary CTT trinucleotide is not avoided, which is consistent with a general view of primed adaptation that involves the recognition of the priming protospacer by the Cascade effector, followed by the recruitment of the Cas3 nuclease-helicase and its processive movement along the DNA away from the priming site in the 3′ to 5′ direction. Such directionality should allow discrimination between 5′-AAG-3′ and 5′-CTT-3′ sequences and will account for observed overall declining gradients of prespacer usage as the distance from the priming site increases.

**FIG 4:**
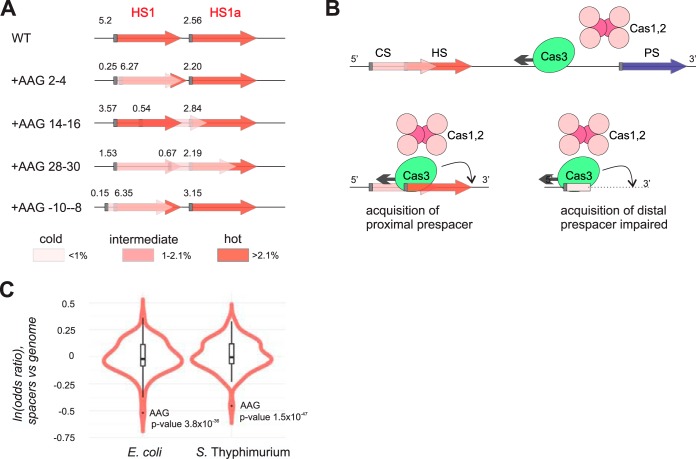
Interdependency of prespacer use during primed adaptation and a possible mechanism. (A) The scheme shows the relative percentages of spacers derived from HS1 and HS1a prespacers in experiments shown in Fig. 3E for cells transformed with plasmids carrying AAG trinucleotides at the indicated positions of HS1. Gray rectangles indicate AAG PAMs; numbers nearby depict the percentage of corresponding spacers (from averaging of two experimental replicas). The insertion of AAG into HS1 decreases its usage efficiency and gives rise to a new prespacer (Fig. 3E). The frequency of HS1a is unaffected by the introduction of the AAG PAM inside HS1 even if the new prespacer overlaps HS1a. The appearance of a new prespacer due to the introduction of a new AAG upstream of HS1 (+AAG −10 to −8) likewise has no effect on acquisition of HS1 spacers. (B) A model describing a mechanism that may account for observed interdependency of prespacer use is presented. Cas3 moves from the priming protospacer (PS) in a 3′ to 5′ direction. Upon encountering AAG trinucleotide, Cas1 and Cas2 use a ruler-like mechanism to extract a spacer in the backward direction. As a result, the efficiency of use of the overlapping prespacer located further downstream is decreased. (C) Violin plots showing the odds ratio of trinucleotides in spacers versus genome-wide frequency in fully sequenced E. coli and *S.* Typhimurium genomes.

A possible mechanistic basis of AAG avoidance in hot spacers is the competition between overlapping prespacers during spacer selection. We observed that for partially overlapping prespacers with AAG PAM, a prespacer located further away from the priming site has no effect on the use of prespacer located closer, while the reverse is not true (Fig. 4A). Such directionality is consistent with a view that the primed adaptation machinery slides in a 3′ direction from the priming site along the fully double-stranded DNA, occasionally recognizes an AAG trinucleotide, and then extracts a spacer-sized fragment located immediately upstream—i.e., opposite to the direction of lateral movement along the DNA (Fig. 4B). According to this model, one would expect that any internal AAG will have the same negative effect irrespective of its position within the prespacer. The unequal effects of AAG trinucleotides placed in the beginning, middle, and end regions of prespacers on adaptation efficiency revealed in our experiments, with much stronger inhibition produced by AAG located in PAM-proximal seed region, require a more sophisticated model and further experiments to explain.

Our results do not allow to distinguish whether interdependency of overlapping prespacer use is due to prespacer interaction with the adaptation machinery *sensu stricto* (i.e., the Cas1-Cas2 complex) or is determined at an earlier stage by Cas3, which may generate substrates for Cas1-Cas2 as it moves away from the priming site (22, 33). Data suggesting that Cas3 may specifically cleave at AAG PAMs have been presented. Also evidence for preferences for AAG PAMs by the Cas1-Cas2 complex both from structural data (34, 35) and analysis of spacers acquired during naive adaptation (15) is available. It is thus possible that Cas3 and Cas1-Cas2 cooperate with each other during primed adaptation, increasing the likelihood of selection of prespacers with AAG PAM, which should have the highest protective effect. The presence of Cas2-Cas3 fusions in type I-F systems supports the idea of such synergy (36). For example, the observed negative effects of internal AAG sequences may be the consequence of Cas3 cleavage at these sites and hindering Cas1-Cas2 access to downstream DNA to begin spacer capture.

The absence or presence of internal AAG cannot be the only determinant of prespacer usage. The sampling frequencies of spacers in our set, which correspond to the same AAG counts in prespacers, differ by about 3 orders of magnitude (see Fig. S2 in the supplemental material). The coefficient of determination from the data presented in Fig. S2 shows that only ∼25% of variability of spacer frequencies acquired during primed adaptation can be explained by the presence of internal AAGs. The rest of the variation must be determined by additional sequence or context-specific effects whose nature is currently unknown.

10.1128/mBio.02169-18.2 FIG S2 AAG influence on prespacer selection. Dependence of log values of observed spacer frequencies (*y* axis) on the corresponding AAG counts in prespacers (*x* axis) is shown. Linear fit to this dependence and the corresponding coefficient of determination (*R*^2^) are shown. The figure shows a large range (up to 10^5^-fold) of prespacer acquisition frequencies corresponding to the same AAG counts, clearly indicating that other variables influence prespacer selection frequency. (The same is also indicated by the *R*^2^ value.) Download FIG S2, TIF file, 1.8 MB. Copyright © 2018 Musharova et al. 2018 Musharova et al. This content is distributed under the terms of the Creative Commons Attribution 4.0 International license.

We used the avoidance of internal PAM signal to assess whether priming may have contributed to acquisition of spacers in natural isolates of E. coli and Salmonella enterica serovar Typhimurium. These two microorganisms contain a virtually identical type I-E CRISPR-Cas system with the same PAM and repeats, but share few common spacers. As can be seen from Fig. 4C, compared to overall genomic frequency, AAG is underrepresented in spacers from CRISPR arrays of fully sequenced E. coli and *S.* Typhimurium isolates, suggesting that priming occurs in natural settings in these bacteria.

## MATERIALS AND METHODS

### Strains and plasmids.

The E. coli DH5α strain was used for cloning. The E. coli strain KD263 (K-12 F^+^ *lacUV5-cas3* a*raBp8-cse1* CRISPR I repeat-spacer G8-repeat CRISPR II deleted) (37) and BL21(DE3) were used in primed and naive adaptation experiments, correspondingly.

In order to create the pG8mut_Km plasmid, a fragment of the pRSF1b plasmid (Novagen) containing a kanamycin resistance gene was amplified with primers kan-fragment forward and kan-fragment rev (see Table S3 in the supplemental material). The amplicon was purified, treated with the EcoRI and BamHI, and cloned into the pG8mut plasmid (23).

10.1128/mBio.02169-18.5 TABLE S3 Oligonucleotides used in this study. Download Table S3, DOCX file, 0.1 MB. Copyright © 2018 Musharova et al. 2018 Musharova et al. This content is distributed under the terms of the Creative Commons Attribution 4.0 International license.

### Library and individual mutant construction.

Plasmid libraries with randomized trinucleotide in HS1 prespacer were obtained by a two-step PCR-based mutagenesis using iProof high-fidelity DNA polymerase (Bio-Rad). In the first step, pG8mut_Km was amplified with forward primer HSRun_for containing three randomized nucleotides inside the HS1 region and reverse primer HSRun_rev complementary to the constant region of HSRun_for. (The list of primers used in this work is presented in Table S3.) Twenty cycles of amplification were performed to generate linearized pG8mut_Km with randomized trinucleotides and short inverted repeats containing sequences of primer complementarity. Completed PCRs were treated with DpnI to eliminate the pG8mut_Km template, and reaction products were purified by the GeneJet PCR purification kit. At the second step, the products of the first amplification reactions were further amplified with primers HSRun_rev and HSRun_add, which contained regions complementary to inverted repeats introduced during the first stage. Five amplification cycles were performed. The products of amplification were purified as described above. Finally, a Gibson assembly cloning kit (New England Biolabs) was next used to generate circular plasmids through recombination between the inverted repeats following the manufacturer’s recommendation. Using the procedure outlined above, six different libraries with randomized nucleotides at positions 2 to 4, 5 to 7, 13 to 15, 19 to 21, 28 to 30, and 31 to 33 of HS1 were generated. The results of Gibson assembly were transformed into DH5α cells by electroporation. At least 2,000 kanamycin-resistant colonies for each library were scraped off the plates and used for plasmid purification by GeneJet plasmid miniprep kit (Thermo Scientific).

Individual AAG trinucleotides were introduced in pG8mut_Km by a standard PCR-based site-specific mutagenesis protocol with primer pairs listed in Table S3.

### CRISPR adaptation and plasmid prespacer and acquired spacer amplification.

For primed adaptation, pG8mut_Km, its derivatives containing individual mutations, or plasmid libraries were electroporated into KD263. For library experiments, at least 2,000 kanamycin-resistant colonies were scrapped off plates for each library and pooled. The resulting cell suspension was diluted with LB to an optical density at 600 nm (OD_600_) of 0.1 and allowed to grow at 37°C in the absence of antibiotic. In experiments with individual plasmids, a single colony was used to inoculate 5 ml LB supplemented with 50 μg/ml kanamycin. After overnight growth at 37°C, an aliquot of culture was diluted 100× with LB without antibiotic, and growth was continued. When cultures reached OD_600_, they were induced by 1 mM arabinose and 1 mM IPTG (isopropyl-β-d-thiogalactopyranoside) at an OD of 0.4. The growth was continued for 6 h.

For naive adaptation, BL21(DE3) cells were electroporated with plasmids pCas1 + 2 (15) and pG8mut_Km. Individual colonies were grown overnight in liquid LB containing 50 μg/ml kanamycin and 50 μg/ml streptomycin. After overnight growth at 37°C, an aliquot of culture was diluted 100× with LB containing 50 μg/ml streptomycin and 0.1 mM IPTG. The growth was continued for 6 h.

Aliquots of cultures were withdrawn immediately before or 6 h postinduction, and total DNA was purified by a Thermo Scientific genomic DNA purification kit. To assess the diversity of HS1 prespacer libraries, the corresponding plasmid region was amplified from 0-h total DNA samples using primers HS1long_for and HS1long_rev. To monitor CRISPR adaptation, CRISPR arrays were amplified from 6-h samples with primers Ec_LDR_F and M13_G8 for DNA from KD263 cultures and moj3-moj4 for BL21(DE3) cultures. Amplicons containing plasmid prespacers and extended CRISPR arrays were gel purified and used to create Illumina sequencing libraries with an NEBNext Ultra II DNA library preparation kit with U5 barcoding. High-throughput sequencing of amplicons was conducted on MiniSeq or HiSeq Illumina machines using the 2 × 150 paired-end mode.

### Bioinformatics analysis.

R script and Bioconductor packages ShortRead (38) and BioStrings (39) were utilized for Illumina reads preprocessing, prespacer and spacer extraction, mapping, and statistical analysis. R package ggplot2 (40) was used for plotting. The following parameters were used: FREDscore for read quality of ≥20, up to 2 mismatches for identification of CRISPR repeats or prespacer flanking regions, and 0 mismatches for mapping. Only uniquely mapped 33-bp-long spacers were taken for further analysis. Circular visualization of plasmid mapping results was done with EasyVisio tool developed by Ekaterina Rubtsova. Odds ratios for each mono-, di-, and trinucleotide were calculated based on Fisher’s test. The odds ratios were calculated for prespacer libraries and acquired spacers or for hot and cold prespacers and/or their flanking sequences.

Spacers acquired during primed adaptation were mapped to the nontarget strand, and log values of their observed sampling frequencies (just sampling frequencies below) were used in the analysis. To decrease noise, the sampling frequencies of reads from different experiment replicas corresponding to same plasmids, which were mapped to same plasmid positions, were averaged. Sampling frequencies corresponding to different plasmids were then normalized to the same mean.

A window of 6 bp in length was slid across 33-bp prespacer sequences and the upstream and downstream prespacer flanking regions of the same length. For each window position, AAGs in the frame were counted, and their means for hot and cold categories (ν_*h*_ and ν_*c*_, respectively) were subtracted. To estimate confidence bounds, it was assumed that the number of counts follows a Poisson distribution, so the standard deviation for the subtracted means was estimated to be $\sqrt{\nu_{h}+\nu_{c}}$.

To additionally assess significance of the AAG position within the prespacer, prespacers were divided into 3 nonoverlapping 11-bp-long regions (upstream, middle, and downstream). For each of these regions, Pearson’s correlation coefficient (*R*) between the number of AAG counts and the corresponding spacer frequencies was calculated. Confidence bounds and *P* values for the obtained correlation coefficients were estimated through Fisher’s *z* transformation.

To assess what fraction of variability in the spacer frequencies can be explained by AAG presence/absence, *R* between the number of AAG counts in the entire spacer and the corresponding spacer frequencies was calculated, from which the corresponding coefficient of determination (*R*^2^) was obtained.
