# Supplementary material for: Avoidance of Trinucleotide Corresponding to Consensus Protospacer Adjacent Motif Controls the Efficiency of Prespacer Selection during Primed Adaptation
Source: mBio. 2018 Dec 4;9(6):e02169-18. doi: 10.1128/mBio.02169-18 (PMC6282206; doi:10.1128/mBio.02169-18)
Supplement: TABLE S1 [file mbo006184199st1.docx]

Table S1.

| Target plasmid | strain | citation | Number of spacersx1000 | Plasmid derived spacers,% | Plasmid PAM bias,% | genome AAG bias,% | Correlation  Pearson  NT/T |
| --- | --- | --- | --- | --- | --- | --- | --- |
| pRSF_G8mut | KD263 | Semenova et al., 2016 | 90,4 | 99 | 98.4 | 91.3 | 0.99/0.99 |
|  |  |  | 140.7 | 99 | 97.7 | 87.3 |  |
| pUC_G8mut | KD263 | Krivoy et al., 2018 | 163.4 | 99 | 98.5 | 74.7 | 0.99/0.99 |
|  |  |  | 106.8 | 99 | 97.0 | 87.4 |  |
| pG8mut_Km | KD263 | here | 1187.8 | 98.5 | 97.7 | 85.5 | 0.95/0.84 |
|  |  |  | 639 | 99 | 97.8 | 87.6 |  |
