# Supplementary figures and images for: Avoidance of Trinucleotide Corresponding to Consensus Protospacer Adjacent Motif Controls the Efficiency of Prespacer Selection during Primed Adaptation
Source: mBio. 2018 Dec 4;9(6):e02169-18. doi: 10.1128/mBio.02169-18 (PMC6282206; doi:10.1128/mBio.02169-18)

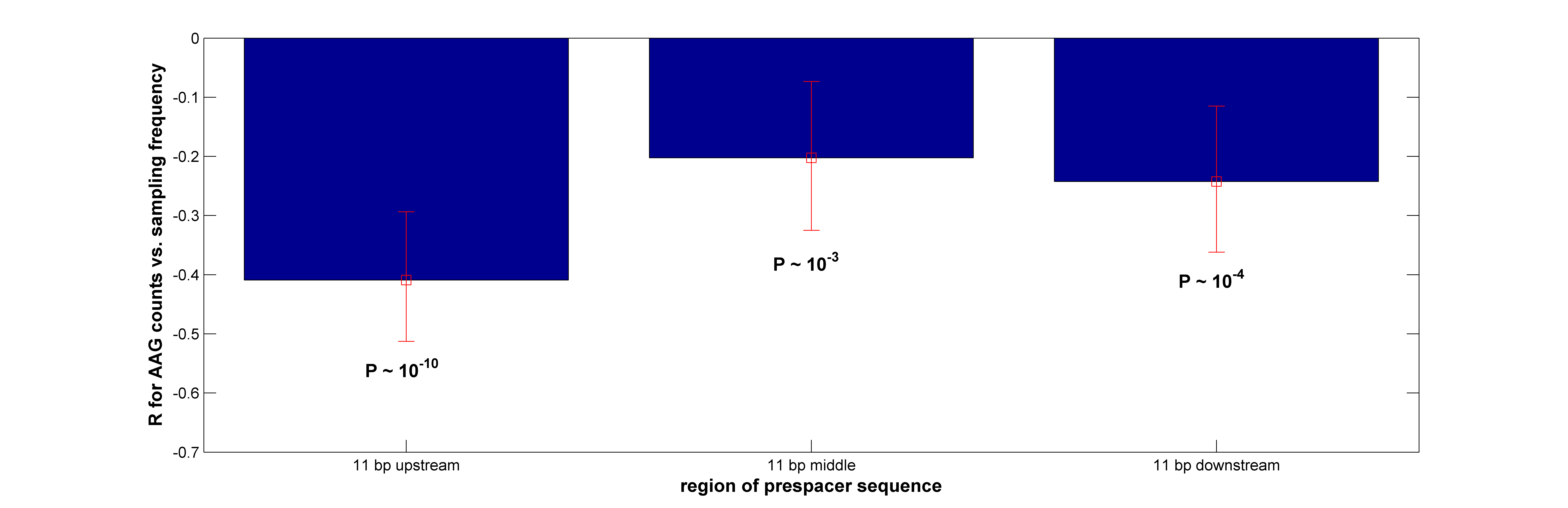

Supplement: FIG S1 [file mbo006184199sf1.tif]

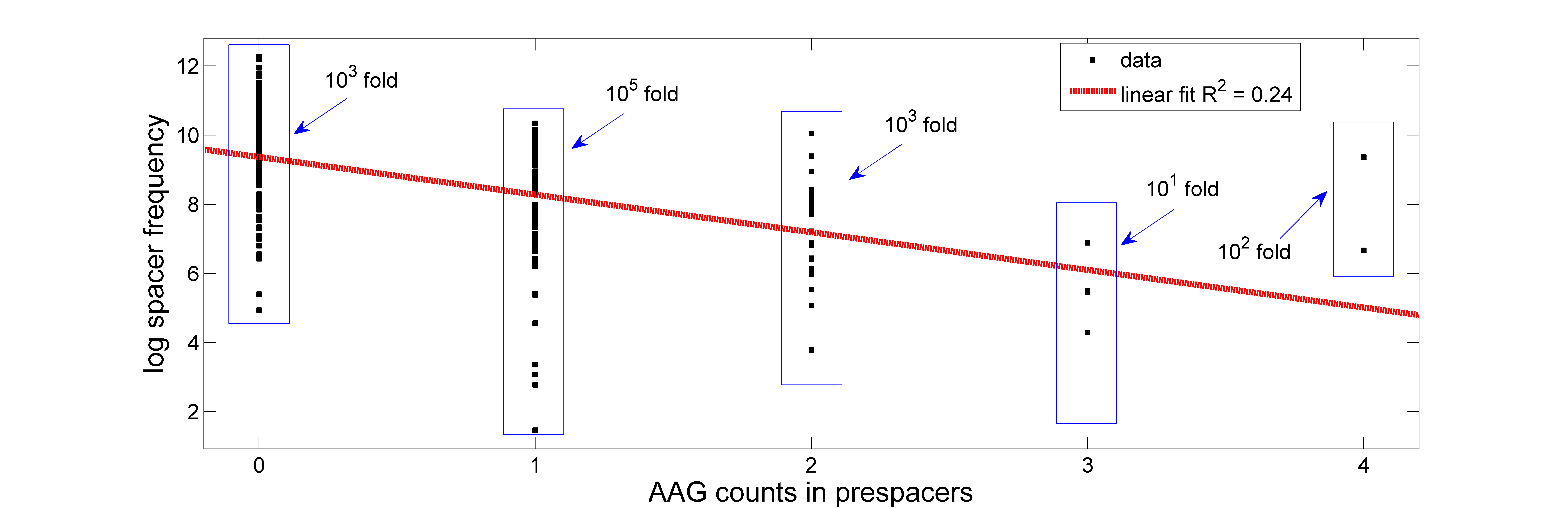

Supplement: FIG S2 [file mbo006184199sf2.tif]
