# Supplementary material for: Avoidance of Trinucleotide Corresponding to Consensus Protospacer Adjacent Motif Controls the Efficiency of Prespacer Selection during Primed Adaptation
Source: mBio. 2018 Dec 4;9(6):e02169-18. doi: 10.1128/mBio.02169-18 (PMC6282206; doi:10.1128/mBio.02169-18)
Supplement: TABLE S2 [file mbo006184199st2.docx]

Table S2.

|  | Spacers mapped | Spacer mapping, % | Strand bias,% | AAG bias,% |
| --- | --- | --- | --- | --- |
| First replicate | 564365 | Genome – 8.8 | 51.5 | 37.7 |
|  |  | pCas1+2 – 89.8 | 44.4 | 49.1 |
|  |  | pG8mut_Km – 1.4 | 50.2 | 46.2 |
| Second replicate | 901327 | Genome – 11.5 | 49.9 | 46.5 |
|  |  | pCas1+2 – 87.6 | 44.8 | 52 |
|  |  | pG8mut_Km – 0.9 | 50.2 | 48 |
