# Supplementary material for: Avoidance of Trinucleotide Corresponding to Consensus Protospacer Adjacent Motif Controls the Efficiency of Prespacer Selection during Primed Adaptation
Source: mBio. 2018 Dec 4;9(6):e02169-18. doi: 10.1128/mBio.02169-18 (PMC6282206; doi:10.1128/mBio.02169-18)
Supplement: TABLE S3 [file mbo006184199st3.docx]

Table S3.

| **Name** | **Sequence (5'-3')** |
| --- | --- |
| kan-fragment_for | AAAAAGGATCCGCAAGTGGCACTTTTCGG |
| kan-fragment_rev | AAAAAGAATTCAAGCGGAAGAGCGCTGC |
| HSRan_for_28-30 | GGAAAACGTTCTTCGGGGNNNAAACTCTCAAGGATCTTACC |
| HSRan_rev_28-30 | CCCCGAAGAACGTTTTC |
| HSRan_add_28-30 | GAAAACGTTCTTCGGGG |
| HSRan_for_31-33 | ATTGGAAAACGTTCTTCGNNNCGAAAACTCTCAAGGATCTT |
| HSRan_rev_31-33 | CGAAGAACGTTTTCCAAT |
| HSRan_add_31-33 | ATTGGAAAACGTTCTTCG |
| HSRan_for_2-4 | CACATAGCAGAACTTTAAAAGNNNTCATCATTGGAAAACGTTC |
| HSRan_rev_2-4 | CTTTTAAAGTTCTGCTATGTG |
| HSRan_add_2-4 | CACATAGCAGAACTTTAAAAG |
| HSRan_for_5-7 | ATAGCAGAACTTTAAAAGTGCNNNTCATTGGAAAACGTTCTTC |
| HSRan_rev_5-7 | GCACTTTTAAAGTTCTGC |
| HSRan_add_5-7 | GCAGAACTTTAAAAGTGC |
| HSRan_for_14-16 | CTTTAAAAGTGCTCATCATTGNNNAACGTTCTTCGGGGCGAAAAC |
| HSRan_rev_14-16 | CAATGATGAGCACTTTTAAAG |
| HSRan_add_14-16 | CTTTAAAAGTGCTCATCATTG |
| HSRan_for_20-22 | AAGTGCTCATCATTGGAAAACNNNCTTCGGGGCGAAAACTCTC |
| HSRan_rev_20-22 | GTTTTCCAATGATGAGCAC |
| HSRan_add_20-22 | GTGCTCATCATTGGAAAAC |
| HS1 long_for | TCAACAGCGGTAAGATCCTTG |
| HS1 long_rev | GCGTCAATACGGGATAATACC |
| M13_G8 | TCCACAGACAGCCCTCATAGTTAG |
| Ec-LDR-F | AAGGTTGGTGGGTTGTTTTTATGG |
| moj3 | GTGGTTTGAGCGATGATAT |
| moj4 | AGTTGGTAGATTGTGACTG |
| HS +AAG 2-4 for | GCAGAACTTTAAAAGAAGTCATCATTGGAAAAC |
| HS +AAG 2-4 rev | GTTTTCCAATGATGACTTCTTTTAAAGTTCTGC |
| HS +AAG 14-16 for | GTGCTCATCATTGAAGAACGTTCTTCGGGGCGA |
| HS +AAG 14-16 rev | TCGCCCCGAAGAACGTTCTTCAATGATGAGCAC |
| HS +AAG 28-30 for | GGAAAACGTTCTTCGAAGCGAAAACTCTCAAGG |
| HS +AAG 28-30 rev | CCTTGAGAGTTTTCGCTTCGAAGAACGTTTTCC |
| HS +AAG -10--8 for | CGCGCCACATAGCAGAAGTTTAAAAGTGCTCAT |
| HS +AAG -10--8 rev | ATGAGCACTTTTAAACTTCTGCTATGTGGCGCG |
| CS -AAG 2-4 for | AAGAACATCCTTTGATCTTTTCTACGGGGTCTGAC |
| CS -AAG 2-4 rev | GTCAGACCCCGTAGAAAAGATCAAAGGATGTTCTT |
